# Supplementary material for: The Impact of miR-155-5p on Myotube Differentiation: Elucidating Molecular Targets in Skeletal Muscle Disorders
Source: Int J Mol Sci. 2024 Feb 1;25(3):1777. doi: 10.3390/ijms25031777 (PMC10855706; doi:10.3390/ijms25031777)
Supplement: Supplementary file 1 [file ijms-25-01777-s001.zip › ijms-2701703-supplementary-figures.pdf]

International Journal of
Molecular Sciences

Supplementary Materials:

Meta-analysis

Ten studies were identified in four conditions related to aging, muscular dystrophies, physical exercise, and skeletal muscle atrophy models. We observed increased expression of this miRNA in a mouse model of atrophy induced by desmin deletion (knockout) (GSE34388) [30]. Meanwhile, miR-155 showed a tendency to increase expression without statistical significance (p-value = 0.077) in Duchenne's muscular dystrophy (GSE1007) [31]. In addition, no difference in the expression of this miRNA was observed in other muscle dystrophy models (GSE5304, GSE1776, GSE12580) [32,33], aging (GSE 25941, GSE674, GSE362) [34–36] and exercise studies (GSE43760) [37] (Supplementary Figure 1, Supplementary Table S2).

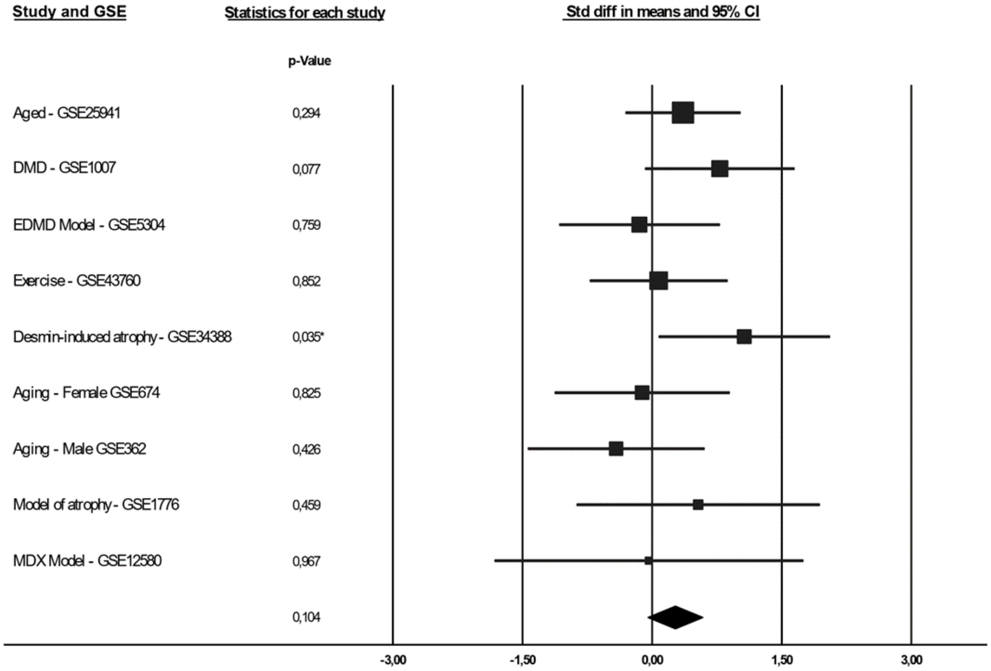

Supplementary Figure S1. Forest plot (random-effects model) demonstrating alterations in the expression of the miR-155 in skeletal striated muscle under experimental conditions or animal models. Squares indicate a relative change in study-specific miR-155 (square size reflects study-specific statistical weight); horizontal lines indicate a 95% confidence interval (CI); and diamonds indicate the summary estimate of the relative change with a 95% CI.

Regulatory gene networks of transcriptional factors and kinases

Our differentially expressed genes were indicated as controlled by a network of transcriptional factors (TF) and kinases using the X2K web tool. These regulatory gene networks of TF and kinases were structured according to the molecule's connectivity, as indicated by network analysis using the Cytoscape program. For data representation, we used the four transcriptional factors with p-values < 0.05; and the top 10 kinases with p-values < 0.05, considering that 68 kinases were indicated as subnetwork regulators.

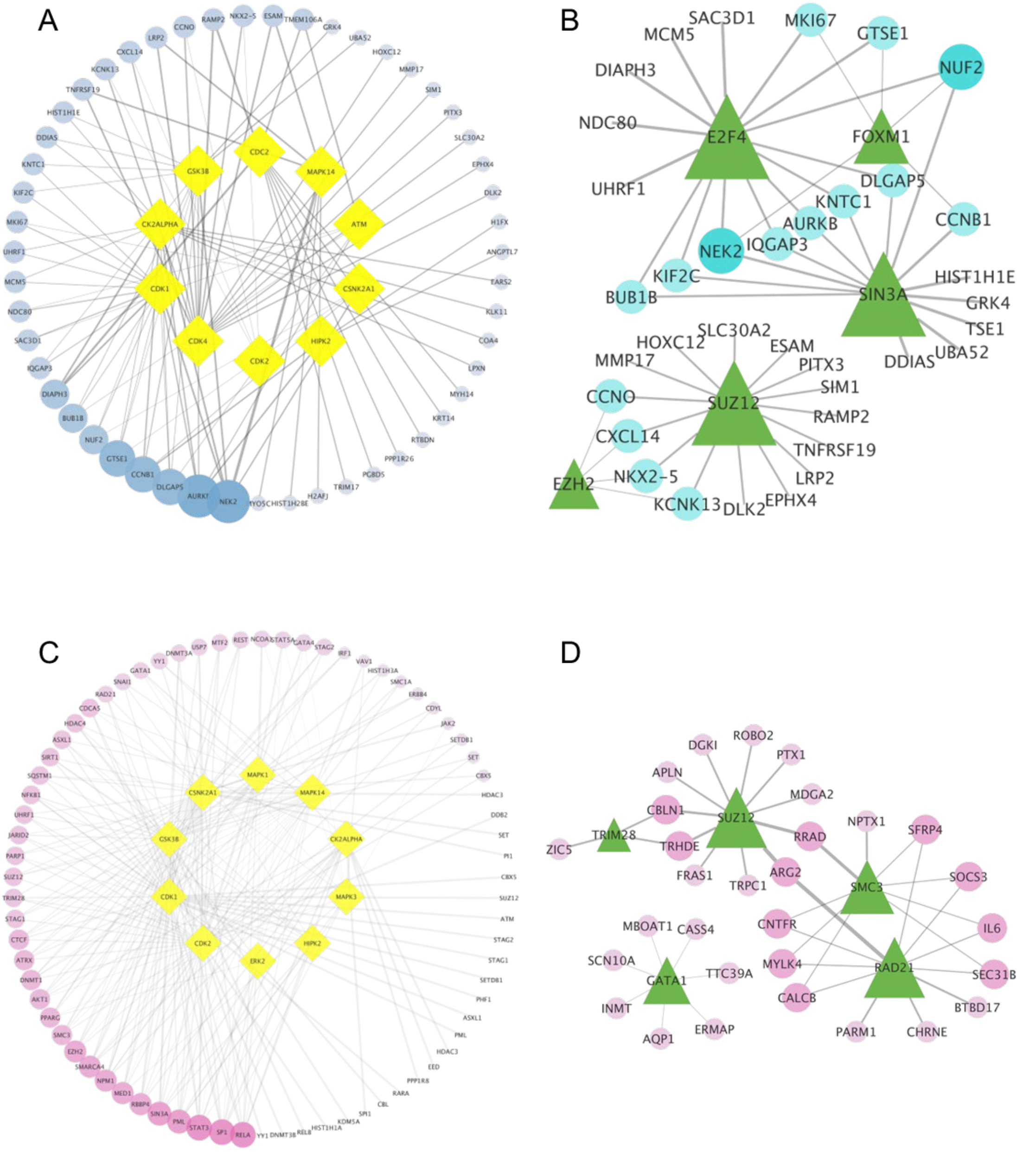

Supplementary Figure S2. Potential transcription factors and kinases that regulate the DEGs of miR-155-treated C2C12 myotubes. Gene regulation network of differentially expressed genes (blue – down-regulated; pink – up-regulated), transcription factors (green triangles), and kinases (yellow diamonds), indicating gene enrichment. The genes (ellipse) and their regulatory kinases or transcription factors are sized based on the connectivity score (highest score → largest size). (A) Top 10 kinases (yellow) regulated by miR-155 that were down-regulated (blue). (B) Top five transcription factors (green) regulated by miR-155 that were down-regulated (blue). (C) Top 10 kinases (yellow) regulated by miR-155 that were up-regulated (pink). (D) Top 5 transcription factors (green) regulated by miR-155 that were up-regulated (pink).

Experimental Design

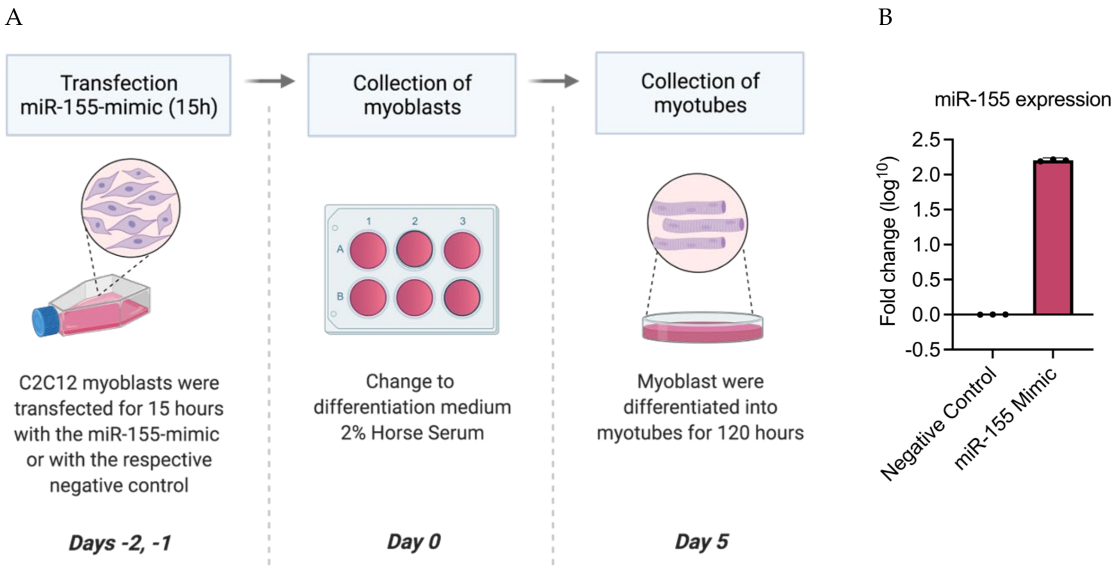

Supplementary Figure S3. A) Experimental design for the functional analysis of miR-155-mimic during myogenesis. After reaching ~ 80% confluence, the cells were transfected for 15 hours with the miR-155-mimic oligonucleotides and their negative control. After transfection, myoblasts were collected. Subsequently, in a 6-well plate, another set of transfected myoblasts was differentiated after changing the growth medium (DMEM + 10% fetal bovine serum + 1% antibiotic) to differentiation medium (DMEM + 2% horse serum + 1% antibiotic) for 120 hours (5 days) and then the myotubes were collected. B) miR-155 expression (fold change [log10]) in myotubes (day 5) treated with miR-155 mimic and negative control.
